# Aging Successfully in the Changing Norwegian Welfare State: A Policy Analysis Framed by Critical Gerontology

**DOI:** 10.1093/geront/gnac177

**Published:** 2022-12-07

**Authors:** Bodil H Blix, Gudmund Ågotnes

**Affiliations:** Department of Health and Care Sciences, Faculty of Health Sciences, UiT The Arctic University of Norway, Tromsø, Norway; Department of Welfare and Participation, and Centre for Care Research, West, Faculty of Health and Social Sciences, Western Norway University of Applied Sciences, Bergen, Norway

**Keywords:** Critical discourse analysis, Critical gerontology, Healthcare policy, Healthy/active aging, Neoliberalism

## Abstract

**Background and Objectives:**

In the 21st century, the future of the Norwegian welfare state is broadly debated. In Norway, as in other countries, concerns regarding the sustainability and affordability of the welfare state in light of the projected population development have been voiced in public and academic discourse, and not least in governmental statements and documents. Because we consider texts, such as government white papers, as both products and producers of discursively based understandings of the social world, a close examination of policy documents can provide insight into the predominant understanding of a distinct phenomenon in a specific society at a particular point in time.

**Research Design and Methods:**

The article is based on a critical discourse analysis of 3 recent Norwegian government policy documents addressing the older adult population.

**Results:**

We demonstrate that prominent ideas from the widely contested successful aging paradigm are embedded and forwarded in current Norwegian policies, where ideas about successful and healthy aging produced and reproduced in the documents frame and shape expectations toward older adults.

**Discussion and Implications:**

We argue that the ideas and ideals of successful aging and neoliberalism in parallel pave the way for changes in the historically generous and comprehensive Norwegian social democratic welfare state. For decision makers, the rhetoric of successful aging that emphasizes activity, productivity, self-reliance, and freedom of choice is undoubtedly more convenient to communicate to the public than explicit arguments for the necessity of downscaling public services.

Norway has a relatively small population of 5.4 million and, similarly to other comparable countries, an aging population. By the end of 2021, 16% of the population was aged 67 or older (Statistics Norway, 2022). The population aged 65 years or older is projected to double by 2075, reaching almost 2 million by 2100 (Statistics Norway, 2020). The Norwegian social democratic welfare state is considered comprehensive and generous (Christensen & Wærness, 2018; Esping-Andersen, 1990), and universalism, the ideal that all health and welfare services should be provided equitably to all citizens regardless of age, gender, ethnicity, financial status, and place of residency, has been a long-standing principle (Anttonen & Sipilä, 2012). Until the early 1980s, there was a broad general consensus regarding the development of the welfare state (Selle, 1993). However, through several reforms in the 1980s and 1990s, the regional and local administrative levels were given more responsibilities in the provision of care for older adults,  primarily in long-term care institutional settings and the home care services (Romøren, 1991), and there was a transformation of orientation from the public sector to the private and voluntary sectors (Rømming, 1999). These reform processes were a result of increased pressure on and concern for the sustainability of the welfare state and devised in parallel with the strengthening of neoliberal ideas and the introduction of New Public Management involving an increased focus on cost-effectiveness (Christensen & Lægreid, 2001; Rømming, 1999). More recently, in line with developments seen elsewhere, it has been argued that yet another shift, from public care services to informal and unpaid caregivers, has occurred (Ågotnes et al., 2021). In the 21st century, the future of the Norwegian welfare state is broadly debated. In Norway, as in other countries (Gusmano & Okma, 2018), concerns regarding the sustainability and affordability of the welfare state in light of the projected population development have been voiced in public and academic discourse, particularly the official or public rhetoric connected to this “problem” (Ågotnes et al., 2021; Blix & Hamran, 2018).

The growing concern for population aging and the sustainability of the welfare state has been accompanied with the conceptualization of “successful aging” (Bulow & Soderqvist, 2014) and, more recently, “healthy aging” (Stephens, 2017). In 2002, The World Health Organization launched its Active Ageing Policy Framework (World Health Organization, 2002), emphasizing “the need to encourage and balance personal responsibility (self-care), age-friendly environments and intergenerational solidarity” and encouraging “[i]ndividuals and families […] to plan and prepare for older age, and make personal efforts to adopt positive personal health practices,” while simultaneously stressing the importance of “supportive environments” (p. 17). The Active Ageing Policy Framework was later replaced by the concept of “healthy aging” through the UN Decade of Healthy Ageing (World Health Organization, n.d.). Through these initiatives, the World Health Organization has framed “healthy aging” as “developing and maintaining the functional ability that enables well-being in older age,” and defining “functional ability” as “determined by the intrinsic capacity of an individual […], the environment in which he or she lives […] and the interactions among them” (World Health Organization, 2020, p. 3).

In this article, we explore how prominent ideas from the successful aging paradigm unfold in current Norwegian policies, and we discuss how associated expectations toward older adults may be linked to specific political and economic assumptions gaining terrain in the changing Norwegian welfare state.

## Successful Aging Through the Lens of Critical Gerontology

As a subfield of gerontology (Holstein & Minkler, 2007), critical gerontology provides a critical perspective both on aging and/in society and on the field of gerontology itself. “[C]ritical gerontology looks inward as well as outward, critiquing the structures, assumptions and practices of mainstream gerontology, along with the sociopolitical environments in which we age” (Ray, 2008, p. 97). Critical gerontology perspectives suggest that the roles ascribed to older adults are shaped by cultural values and norms, and by policies and economics (Rozanova, 2010). In this article, critical gerontology provides us with a double gaze through which we can both “look inward” to explore ideas about successful aging, and “look outward” on how these ideas operate in policies aimed at the aging population in the changing Norwegian welfare state.

“Successful aging,” as a theoretical advancement in the field of gerontology, developed as a response to earlier literature representing apocalyptic descriptions of aging (Martin et al., 2009) based on the assumption of decline, degeneration, and withdrawal as normal features of aging (Johnson & Mutchler, 2013). The notion of successful aging developed as part of a movement toward “a more positive gerontology” focused on “the significance of engagement with life and participation in socially valued roles” for older adults (Johnson & Mutchler, 2013, p. 94). Among the most prominent advocates were Rowe and Kahn (1997, 1998), who associated successful aging with avoidance of disease and disability, maintenance of cognitive and physical function, and engagement in social and productive activities. According to Rowe and Kahn, successful aging is “largely under the control of the individual” and can be attained “through individual choice and effort” (Rowe & Kahn, 1998, p. 37).

The new and “positive gerontology” represented a shift in the fields of gerontology and geriatrics from treatment to prevention and toward the individualization of the responsibility for obtaining and maintaining health/success in old age (Bulow & Soderqvist, 2014). Accordingly, policies shifted in many countries from focusing on care for older adults to promoting prevention, participation, and the maintenance of older adults’ health to reduce the potential burden on health and welfare systems (Stephens, 2017).

Although quickly gaining a prominent position in the gerontology literature, the concept of successful aging has since been widely contested (Stephens, 2017). In a systematic review of journal articles published between 1987 and 2013, Martinson and Berridge (2014) identified four categories of critiques: *Add and Stir*; *The Missing Voices*; *Hard Hitting Critiques*; and *New Frames and Names*. Critiques falling into the category of Add and Stir suggested less strict criteria for successful aging, while The Missing Voices advocated for including diverse older adults’ perceptions of successful aging. In the category of Hard Hitting Critiques were those outright rejecting successful aging as a paradigm, calling for less individualistic and more inclusive frameworks, and the necessity of attending to the structural contexts of aging. Finally, the New Frames and Names category argued for alternative and more holistic models. In summary, successful aging has been criticized for ignoring the impact of gender, class, and race (Calasanti & Slevin, 2001; Holstein & Minkler, 2003). Moreover, critiques have noted that rather than countering ageism, the successful aging framework might increase it by imposing on older adults the fear of aging and a sense of failure to age successfully (Calasanti, 2015). Critiques have also noted that the ideal of successful aging may be both ageist and ableist (Gibbons, 2016), with others pointing out the close association between successful aging and neoliberalism, arguing that both paradigms represent a shift toward a focus on individual responsibility and failure to recognize those who are physically or socioeconomically disadvantaged and therefore unable to “age successfully” (Rubinstein & de Medeiros, 2014; Timonen, 2016).

## Materials and Methods

### Methodology: Critical Discourse Analysis and Critical Gerontology

Texts, such as government white papers, both actively produce and are products of discursively based understandings of aspects of the social world (Cheek, 2004). Statements, and the relationships among them, create and sustain understandings and “truths” while also containing (often telling) silences. Close examinations of government white papers can provide insight into the predominant understandings of a distinct phenomenon in a specific society at a particular point in time. Thus, we consider a discourse analysis of Norwegian government white papers an appropriate approach to inquire into contemporary understandings of aging and old age in Norwegian health and care policies, and perhaps even in contemporary Norwegian society. We consider this endeavor as relevant and significant given the important role of Norwegian white papers, not only for policy development, but also for the organization and reform of welfare services (Jacobsen, 2015).

This article is based on a critical discourse analysis (CDA) of a strategic sample of three recent Norwegian government white papers addressing the older adult population. Fairclough (2001, p. 122) noted that CDA is “as much theory as method—or rather, a theoretical perspective on language and more generally semiosis.” Moreover, he emphasized its potential for studying “‘interdiscursivity’ of a text […] which genres, discourses and styles it draws upon, and how it works them into particular articulations” (p. 125). CDA “examines the ways in which language produces and moderates social and psychological phenomena” with a particular attention on “language as a power resource” (Mullet, 2018, p. 116). Hence, CDA provided a suitable analytical framework for inquiring into the assumptions about older adults and old age, and the political and economic assumptions produced and reproduced in the documents. The following research question guided the first step of our analysis: What expected practices or individual behaviors among older adults are produced and/or reproduced in the documents? The next step of our analysis was guided by the following research question: What values and norms, and political and economic assumptions, are the documents products and producers/reproducers of?

### Material

The included white papers were launched within the last 4 years (2018–2021). All documents are Reports to the Storting (Parliament), that is, the government’s report to the Parliament on the work conducted in a particular field; these often form the basis of a draft resolution or bill at a later stage (cf. https://www.regjeringen.no/en/find-document/white-papers-/id1754/). The documents are presented in Table 1.

**Table 1.** Included White Papers

Document	Responsible ministry	Year published	Number of pages	Short title used in in-text quotes
Meld. St. 15 (2017–2018) Leve hele livet—En kvalitetsreform for eldre [A Full Life—All Your Life—A Quality Reform for Older Persons]	Ministry of Health and Care Services	2018	184	A full life
Meld. St. 19 (2018–2019) Folkehelsemeldinga—Gode liv i eit trygt samfunn [Public Health Report—Good Lives in a Safe Society]	Ministry of Health and Care Services	2019	204	Public health report
Meld. St. 14 (2020–2021) Perspektivmeldingen 2021 [Long-term Perspectives on the Norwegian Economy 2021]	Ministry of Finance	2021	308	Long-term perspectives

The selection of documents for analysis was based on recency and relevance, and the authors’ familiarity with the field. The first document (Ministry of Health and Care Services, 2018) was included because this was the most recent government white paper exclusively addressing old age and older adults. The document was launched by the government as “a quality reform for older adults,” as stated in the document title. The second document (Ministry of Health and Care Services, 2019) was included because it addressed older adults and old age within the broader frames of national public health policies. The third document (Ministry of Finance, 2021) is a type of policy document launched every fourth year presenting the government’s perceptions of future challenges and their plans to encounter them. This document was included because it addressed older adults and old age within the frames of the broader national policies from a long-term perspective.

### Critical Discourse Analysis

In the first step of the analysis, we read the included documents to obtain an overall impression. Next, the first author scanned the documents for segments addressing aging/old age/older adult population. This process was necessary because this topic was only one among many addressed in two of the included documents (Long-term perspectives and Public health report). In the next step of the analysis, the first author read the documents using the first research question as an analytical lens; What expected practices or individual behaviors among older adults are produced and/or reproduced in the documents? Relevant passages of the text were highlighted, and these passages were presented to and discussed with the second author. The next step of the analysis was guided by our second research question; What values and norms, and political and economic assumptions, are the documents products and/or producers of? As for research question 1, the first author conducted the initial analysis and presented and discussed the findings with the second author. In our discussion of the initial findings, we paid particular attention to similarities, differences, and conflicts among statements within and between the included documents. The two authors’ diverse theoretical and methodological expertise provided rich opportunities for investigator triangulation to reduce the risk of biased interpretations (cf. Polit & Beck, 2012, p. 593). In combination, the first author’s position in gerontology and the second author’s position in social sciences provided us with the abovementioned “double gaze” of critical gerontology. The dialogue between our respective perspectives in gerontology and social sciences throughout the analysis is also in line with Fairclough’s (2001) ambition for CDA to be a transdisciplinary endeavor.

Quotes from the included documents were translated from Norwegian to English by the first author. The authors take responsibility for any misrepresentation in the translated quotes. Full-text versions of the included documents are available in Norwegian only, although short versions of two of the documents are available in English (Norwegian Ministry of Finance, 2019; Norwegian Ministry of Health and Care Services, 2018). While we acknowledge that using full-text documents available in Norwegian limits the transparency of our analysis for an international readership, we strongly believe that excluding policy documents from countries other than those with English as an official language would be a limitation to the development of the international gerontology knowledge base.

## Results

The first step of our analysis focused on the expected practices and individual behaviors among older adults produced and reproduced in the documents, that is, the communicated ideals of how to age well. This step of the analysis resulted in the three interrelated imperatives: *Be physically active and healthy! Be self-reliant!* and *Be productive!* The second step of our analysis focused on the political and economic assumptions produced and reproduced in the documents. This step of the analysis resulted in the imperative *Be a conscious consumer rather than a passive care recipient!*

### Be Physically Active and Healthy!

Staying physically active is partly communicated as a goal of its own, as a lifelong endeavor, and partly as the core to the public health policy aimed at the general population. Moreover, physical activity is presented as “a natural choice,” implying that refraining from being physically active is a deviation from the norm: “The vision is physical activity being a natural choice for everybody throughout life” (Public health report, p. 116).

With respect to the older adult population, the imperative to stay active and remain healthy is not only a goal in itself; it is also closely linked to the imperative to stay self-reliant (to which we will return shortly), which leads, in combination, to reduced needs for help and support from others:

Research on physical activity and health throughout the life course indicates that those who are physically active have far better health than those who are not. Physical activity prevents disease and illness, improves physical functioning, and gives more healthy years. This is important to stay self-reliant for as long as possible, and thereby reduce the need for care services (Long-term perspectives, p. 283).

Other statements in the documents indicate that the imperative to stay healthy is not necessarily the end goal for the older adult population, despite the cumulative effects as stated in the abovementioned quote. A healthy older adult population is rather presented, among other efforts such as the implementation of assistive technology, as important to reduce the pressure on the health and care sector:

If the health of the older adult population is improved or we manage to take measures to reduce the need for health and care services, e.g., by using various forms of assistive technology, the growth in the [health and care] sector will decelerate (Long-term perspectives, p. 115).

Here, the older adult population is explicitly highlighted as a “target population” to fulfill the aim of decelerating the growth in the health and care sector. As such, they represent both “the problem” and “the solution.” Moreover, the imperative of staying healthy is not only related to consequences for the health and care services; it is also closely linked to the sustainable development of society at large:

Good health and quality of life is first and foremost important for the individual but must also be considered in light of the need for a sustainable development of society. A population with good health is an important resource for work life and society at large (Public health report, p. 191).

Active and healthy aging is, as such, presented both as pragmatic and moral entities, that is, both as a goal in itself (“important for the individual”) and as a means to reduce pressure on the health and care services and safeguard the sustainability of society at large (“the need for a sustainable development of society”). In both entities, staying active and staying healthy is presented as an individual responsibility, and policies should first and foremost facilitate that individual older adults take this responsibility: “Active aging through taking responsibility for one’s own health and managing on one’s own, is both an ideal for the individual and a political strategy” (A full life, p. 83).

### Be Self-Reliant!

As seen, staying active and healthy is presented as generally beneficial, both for the older adult and society. Active aging is, as such, connected not only to better health for the older adult but also to her ability to be self-reliant and independent: “The goal is that every single human should use their resources and maintain self-reliance for as long as possible” (A full life, p. 25). Especially for the public care sector, these benefits are presented as tangible: a more self-reliant older adult population is assumed to alleviate the pressure on care services: “An important area for prevention is older adults’ need for care. Public expenses will depend on how well-suited older adults are to take care of themselves along with families and communities” (Long-term perspectives, p. 283).

The imperative of being self-reliant is thus portrayed not only as a moral project (as good for the older adult) but also as a form of moral *duty*: the older adult has a responsibility to remain self-reliant to contribute to the sustainability of the welfare state. Consequently, it is suggested that, in the future, well-being in old age will to a lesser extent be a responsibility for the welfare state and to a greater extent an individual responsibility: “…everybody should be prepared to take on greater personal responsibility in the future” (Long-term perspectives, p. 283).

The individual responsibility is extended not only with respect to scope (*what* the individual is expected to take responsibility for) but also with respect to time (*when* the individual should take responsibility). The policy documents encourage “midlife older adults” to “plan for their own old age,” for example, by “investing in friends and social networks” and the “accommodation of one’s home” (A full life, pp. 72ff) without problematizing the inequitable distribution of resources (such as housing and social networks) in the heterogeneous populations of “midlife older adults” and older adults. Hence, “increased prosperity” is postulated as the norm for the future older adult population:

Increased prosperity makes older adults better suited to accommodate their residence in order to be prepared for a situation with impaired health, and to pay for care. To facilitate good care services for those in need, it is important that people in mid-life and older adults take greater responsibility for providing suitable housing and prepare for a situation with increased need for help (Long-term perspectives, p. 283).

While “the future older adult” is postulated as increasingly prosperous and resourceful, the role of “the future public sector” is portrayed as having the role of informing and facilitating “the future older adult population” to realize its inherent potential:

Faced with demographic changes, it is necessary to challenge the population to take on greater responsibility for the planning of old age. [The national program for an age-friendly Norway] will launch an information campaign focusing on: the accommodation of own residence, investing in friends and social networks, maintaining best possible functional ability through an active life (A full life, p. 12).

### Be Productive!

As seen in the previous themes, older adults are expected to be active, healthy, and self-reliant leading to better outcomes for society and the older adult population themselves, in part because it will alleviate a strained health and welfare system. Moreover, an active, healthy, and self- reliant older adult population is also considered an  untapped resource for society: “Active aging is about considering older adults as a resource for society and facilitating participation and involvement. An age-friendly society involves measures to stimulate activity and coping” (Public health report, p. 20).

The current and coming generations of older adults are portrayed as healthier and more active compared to previous generations and are encouraged and expected to contribute to society accordingly. The “new” older adults have, it is argued, other capabilities and more opportunities than previous generations related to the longevity of their cohort but also the availability and capacity of networks: “Today’s and tomorrow’s older adults live longer, and some have  20 and 30 years as retirees ahead. Many live active lives with good health and quality of life, and participate actively in society, both locally and centrally, in families, organizations, work life and cultural life” (A full life, p. 31).

Coming generations of older adults are also expected to contribute to society through prolonged work life: “More healthy years will make it possible for older adults to remain in work life for longer” (Long-term perspectives, p. 190). By remaining economically productive citizens, older adults could and should contribute to a sustainable society both through reducing pension expenses and contributing to economic growth:

The high level of employment in Norway has been the basis for the development of good and universal welfare arrangements. […] Gradually, there are fewer in the workforce per retired person in Norway. Therefore, a high level of employment in the older population is important (Public health report, p. 159).

To safeguard the sustainability of the welfare state, mobilizing more labour, keeping the average work hours high, and enabling older adults to stay longer in work life is necessary (Long-term perspectives, p. 97).

The imperative to be productive includes expectations for older adults to contribute with unpaid and voluntary work, both within their own families and in communities, thus taking advantage of the capabilities and opportunities available for this particular cohort: “The Senior resource. [The program for an age-friendly Norway] is about using the older adult resource in various organizations in public and private sectors, and in business development” (A full life, p. 76). Additionally, older adults are expected to be coproducers of future services addressed at older adults: “Co-creation is emphasized as important processual measures to create future local communities and services” (A full life, p. 171). In that sense, older adults are responsible not only for themselves and their communities, but also for the services’ “successfulness.”

In a preliminary conclusion, much attention is paid to how older adults (and their families) should be “active,” “participants” and “coproducers” in society, local communities, work life, and services. Less attention, however, is paid to how the imperative to be productive, both through paid and unpaid work, relates to variation and diversity of interest, capabilities, and resources among an (increasingly) heterogeneous older adult population.

### Be a Conscious Consumer Rather Than a Passive Care Recipient!

Demographic aging, phrased in the documents as “increased longevity,” is a premise for the imperatives described above; because of increased longevity, healthy, active, and independent aging is exceedingly important. Interestingly, increased longevity is further presented as a result of society’s collective efforts and a product of a well-functioning welfare state: “Longevity and more older adults are positive. It is a result of society’s efforts to take care of the population’s health and living conditions” (Public health report, p. 75).

As such, “increased longevity” is presented both as a product of a thriving and well-functioning society and as a resource that could and should further benefit society. Simultaneously, the solution to the challenges following this collective success, that is, “more people facing health challenges associated with old age,” “increased expenses for the health and care sector,” and “increased pension expenses for the state” (Public health report, p. 75), are, as we have seen, presented as an individual responsibility to be active, healthy, self-reliant, and productive. In combination, a turn toward individual responsibility is related to the presentation of the older adult as a consumer, emphasizing independence and autonomy as opposed to collaboration and coproduction. The older adult of today (and certainly tomorrow) *could and should* be a conscious consumer rather than a passive care recipient. The imperative of a conscious consumer is expressed through a discourse of “freedom of choice”:

A Full Life is a reform for increased freedom of choice. It should give the individual increased opportunities to choose service provider (who), to contribute to the content of the services (what), in which way the services are provided (how), and in which place and time the services are provided (where and when) (A full life, p. 10).

The shift in the conceptualization of older adults from care recipients to conscious consumers also has implications for the role of the welfare state and the public sector. Her role as a care provider is replaced by the role of a facilitator, whose primary function is to remove barriers for older adults’ activity, self-reliance, productiveness, and choice: “The government’s strategy for an age-friendly society emphasizes the value of healthy and active aging, and the need to remove unnecessary barriers, so that older adults more easily can participate in work and society at large” (Public health report, p. 75).

While the older adult is presented as a conscious consumer, and the welfare state is presented as a facilitator, the public health and care services become gatekeepers and protectors of the sparse welfare state’s resources: “…the health and care services should assess the individual’s needs and potential for rehabilitation and self-care before initiating measures compensating for loss of functional ability” (A full life, p. 21). Interestingly, potential paradoxes arising from the individual’s freedom of choice, for example, with respect to the responsibility to protect the welfare state’s resources by aiming for rehabilitation and self-care rather than more costly services, are largely bypassed in the documents. Rather, moderation at the service level is presented as beneficial for all parties:

The public health policies and the health and care services should contribute to everyone having the opportunity to live throughout life, continuing to participate in family life, cultural and social life, and having a meaningful existence in companionship with others, for the benefit and joy of society (A full life, p. 31).

## Discussion

We believe that discourses on aging influence both how individuals think about aging and how societies structure their social institutions, thereby sustaining or altering power relations (Minichiello et al., 2005). We agree with Rudman (2006) that discourses do not necessarily determine how individuals act and think, but “they provide morally-laden messages that shape possibilities for being and acting” (p. 184). Through our analysis of three recent Norwegian policy documents addressing the older adult population, we identified four interrelated imperatives. The first three imperatives, *Be physically active and healthy! Be self-reliant!* and *Be productive!* are closely associated with core ideas in the “positive gerontology” in which the ideal of successful aging has a prominent position. These imperatives partly correspond with what Timonen (2016, p. 7) termed “The holy trinity,” that is, the expectation that older adults should actively engage in preserving and enhancing independent living skills, stay in paid employment, and remain or become socially productive. The fourth imperative *Be a conscious consumer rather than a passive care recipient!* follows from the initial three imperatives and is closely associated with the neoliberal ideals of marketization and increased involvement of families and civil society with the ultimate goal of a less comprehensive welfare state. Within this imperative, the rhetoric around older adults as coproducers in local communities and services is less prevalent. Even though “older adults as consumers” and “older adults as coproducers” are significant and competing discourses in the Norwegian public discourse (Askheim et al., 2017), we argue that the former has precedence in our material. Core to all four imperatives is the individualization of responsibility and the implicit assumption that people, of all ages, are equitably positioned to take on this responsibility.

As noted by Rudman (2006), discourses can influence the policies that respond to perceived and actual problems. In our material, two parallel discourses aiming at policy changes were identified: one in which the aging population is the problem, and one in which the individual older adult is the solution. At first glance, these two discourses may appear contrasting. However, following closer inquiry, it becomes clear that the two discourses are closely interconnected and mutually reinforcing. The close kinship between successful aging and neoliberalism is based in the individualization of responsibility core to both ideologies. As noted by the abovementioned Hard Hitting Critiques (Martinson & Berridge, 2014), successful aging is instrumental for drawing attention to individualized responses to aging, rather than the various structural factors associated with and affecting population aging (cf. Asquith, 2009). The two parallel discourses identified in the documents are akin to what Timonen (2016, p. 3) referred to as “the paradox of turning the problem into the solution.” We consider the individualization of responsibility as the final step in a series of Norwegian health and care policy reform processes pushing the responsibility for older adult care downward, from the state to municipalities (Rømming, 1999); from the public sector to the private and volunteer sectors (Blix & Hamran, 2018; Skinner et al., 2018); and from the public care services to families (Ågotnes et al., 2021). Mikkelsen (2017, p. 647) has noted a similar tendency in Denmark, where “optimistic discourses on aging are closely tied to wider political strategies for minimizing the budgetary strain on public health systems and managing the aging population.”

Another and related evident duality is the representation of healthy, independent, and productive aging both as beneficial for the individual and society (the pragmatic foundation) and as an individual duty (the moral foundation). This duality remains, however, somewhat ambiguous and obscure—shifting from one statement to the next, within and between the included documents. As such, the documents represent what Dahl (2017, p. 38) termed “a new paradigm of aging that foregrounds the active and healthy old person” in which “an active senior becomes a moral obligation rather than a choice.” These two parallel representations of aging and older adults correspond with Timonen’s (2016, p. 87) two different yet closely related conceptualizations of “model aging.” On the one hand, such models “*model* (verb) old age and the people who are defined as old,” while on the other hand, as a result of the first, the “different *models* (noun) of what are good, appropriate, proper ways to age” come to existence. We share Mikkelsen’s (2017, p. 653) concern that if a society’s expectations toward older adults are internalized by its citizens, it may, in turn, result in those who for various reasons are incapable of living up to such expectations being labeled “morally flawed.” Thus, it may be argued that the moral foundation of the representations of older adults in Norwegian policy documents has an inherent potential for ageism: “if the individual is responsible for his/her success, then the ageism that would befall those who do not age successfully is justified” (Calasanti, 2015, p. 1095). In line with the abovementioned Missing Voices critiques of successful aging (Martinson & Berridge, 2014), we are concerned that the silencing of the fact that the heterogeneous older adult population is inequitably positioned to pursue the imperatives produced and reproduced in the Norwegian policy documents serves to further marginalize older adult individuals and groups with limited access to resources such as money, family, social networks, housing, and outdoor spaces. As such, our concern is that the policies create “a class of those who age unsuccessfully” (Rubinstein & de Medeiros, 2014, p. 38), while simultaneously waiving responsibility for the class created by the very policy. Moreover, we are concerned that the focus on individual choice and behaviors of older and younger older adults draws attention away from a life-course perspective on inequities in health. As noted by Asquith (2009, p. 256),

the profits of good social health are not generated out of the changes in the last years of an individual’s life. Rather, good social health is generational; it requires good health patterns to be initiated in the first age, not the third or fourth age.

A third duality identified in the documents is related to freedom of choice. On the one hand, and in line with neoliberal ideology, older adults are represented as conscious consumers expected to make informed choices about services “consumption” (cf. the who, what, how, where, and when of services provision, A full life, p. 10). On the other hand, the public health and care services are positioned as gatekeepers aiming for the provision of services at the lowest level of effective care (cf. the request that services “should assess the individual’s needs and potential for rehabilitation and self-care before initiating measures compensating for loss of functional ability,” A full life, p. 21). This implies that perhaps “the freedom of choice” is not so much about the “what,” “how,” “where,” and “when,” but rather about the “who,” that is, the freedom to choose between different service providers. Although not stated very explicitly, this indicates a political shift toward a health and welfare system with a greater involvement of private and for-profit service providers in older adult care than what has, so far, been the case in the Norwegian welfare state. Moreover, the documents leave individual older adults with limited freedom to choose *not* to be active, healthy, self-reliant, and productive. Rather, it is assumed that older adults both desire and have the “potential” to age successfully, making the role of the public services both a gatekeeper and a coach; keeping expenses at a minimum by harnessing and releasing the potential of the individual older adult. Mikkelsen (2017, p. 655) noted that, “the paradox of neoliberalism […] is that while it attempts to promote various forms of free choice, […] its success relies on extensive and creative strategies of governance,” and the core of these strategies is the “politics of potentiality.” In the Norwegian policy documents, the “surplus of potential” is represented as increasing through the portrayal of the “new” older adults as having capabilities and opportunities previous generations did not have. In other words, while the “problem”—that is, the older adult population—is growing, so is the solution: the surplus of potential. Within this scenario, we see the contours of a new and less comprehensive welfare state: facilitating, coaching, and perhaps even requiring, individual responsibility for healthy, active, productive, and self-reliant aging.

## Conclusion

This study was based on a limited sample of policy documents, and our analysis was informed by the perspective of critical gerontology. This perspective makes some aspects more visible, while other aspects remain unexplored. Our results should be read considering these limitations. The documents included in the analysis were recent and, thus, considered to represent current Norwegian policies in this field.

Our analysis demonstrates that prominent ideas of successful aging are alive and kicking in recent Norwegian policy documents addressing the older adult population. We argue that the ideas of successful aging have gained renewed interest following increased concern among policymakers for the economic consequences of population aging. Hence, in Norwegian policy, successful aging is not (solely) a goal in itself; rather, it is a means to safeguard the sustainability of the Norwegian welfare state through the individualization of responsibility and the redefinition of the public health and care services’ role in older adult care. As such, we argue that the ideas of successful aging and neoliberalism in parallel pave the way for changes in the historically generous and comprehensive Norwegian social democratic welfare state. For decision makers, the rhetoric of successful aging, emphasizing activity, productivity, self-reliance, and freedom of choice, is undoubtedly more convenient to communicate to the public than explicit arguments for the necessity of downscaling public services. Nonetheless, as noted by others, “the ageing population problem is institutional in nature, its resolution will not be found in individual answers” (Rottier & Jackson, 2003, p. 170). We would add that the sustainability of the welfare state cannot be solved solely by governing individual older adults’ behavior.
